# Editorial: Structural studies of bacteria and viruses

**DOI:** 10.3389/fmolb.2025.1605596

**Published:** 2025-04-30

**Authors:** Annalisa Pastore

**Affiliations:** ^1^ The Wohl Institute, King’s College London, London, United Kingdom; ^2^ Elettra Sincrotrone Trieste, Basovizza, Italy

**Keywords:** bacteria, virus, protein dynamic, pathogen biology, host interaction

The nearly 2 years of secluded life imposed by the COVID-19 pandemia have probably made us all much more aware and sensitive than before to infections and their consequences: spread of the coronavirus emerged in Wuhan, China in December 2019 caused the acute respiratory syndrome coronavirus 2 (SARS-CoV-2) in the entire world and infected more than 88 million people, causing over 1.9 million deaths worldwide (WHO, 2020). In addition to mortality and health problems, the strict confinement, lockdown, and travel restrictions imposed by the pandemia in the attempt to reduce the spread of the contagion have resulted in an uncountable number of socio-economic implications (Mara et al., 2025). As a consequence of the non-trivial impact of COVID-19, new research has been dedicated to the study of viruses, boosting fields beyond that of COVID-19 and more in general of all pathogens and ways to counteract their harmful effect.

In the present Research Topic, we grouped articles that address different aspects of the structural biology of viruses and bacteria. Six papers appeared in Frontiers in 2022, three concerning viral proteins, and three more which concern bacterial proteins.

In the paper by Malär et al., the authors used solid-state NMR to experimentally characterize the dynamics of the 3.5 MDa hepatitis B virus (HBV) capsid, assembled from 240 copies of the Cp149 core protein. By comparing the NMR relaxation parameters *T*_*1*_ and *T*_*1ρ*_ for the capsid protein, used as detectors of dynamics in the nanosecond and microsecond timescale, with values obtained from a 1 microseconds Molecular Dynamics trajectory, the authors could reconstruct the dynamics of the system showing a strong complementarity between the two methods.

In Chen et al., the authors reported in the N-terminal RNA-binding domain (NTD) of the coronavirus N protein a potential allosteric site (Site 1) that modulates its conformation and identified by PARS, an online allosteric site predictor. N is a nucleocapsid protein that forms an oligomer and facilitates binding with the viral RNA genome. 5-hydroxyindole was proposed to target Site 1 based on molecular docking and, based on this knowledge, four 5-hydroxyindole derivatives were designed and synthesized. Two of these 5-hydroxyindole compounds with higher hydrophobic R-groups were shown by Small-Angle X-ray scattering (SAXS) to mediate the binding between the N- and C-terminal domains and promote further oligomerization of the whole N protein while forming a hydrophobic cavity, which mediates the interaction between the two domains. The study thus indicated an allosteric binding pocket that induces aggregation of the N protein and that may be targeted by anti-COVID drugs.

In the review by Hsu et al., the authors summarized recent structural and mutational characteristics of the RNA binding domain of the human coronavirus spike protein and of its interaction with specific human cell receptors and analysed the structural requirements and plasticity of the RNA binding domain. The study is interesting because the stability of the spike protein, affinity toward receptor, virus fitness, and infectivity are the factors controlling the viral tropisms. The authors argued that structural information about the spike protein and the receptors of human coronaviruses are the key to understand the molecular mechanism of host-microbe pathogenesis and can be used as the starting point to develop effective drugs to control infection in future coronavirus outbreaks.

Ghosh et al. studied the protein 3-oxoacyl-[acyl-carrier-protein] reductase (FabG), a key component in the type II fatty acid synthase (FAS II) system from the gram-positive bacterium *Streptococcus pneumoniae*. FabG is responsible for various infections caused by the bacterium, and its antibiotic resistance presents significant challenges to medical treatment and is thus a target for new anti-streptococcal antibiotics. The authors solved the SpFabG structure by X-ray diffraction at 2 Å coadjuvated by AlphaFold models for molecular replacement. They also suggested that dimers rather than the crystallographic tetramer could be the active conformation in solution although formation of the tetramer of is important for the enzyme activity. By comparing the structures of FabG homologues, the conserved tetramerization site was identified. Finally, a thorough pharmacophore-based virtual screening study using a vast library of compounds (533,600 small molecules), supplemented by molecular docking and binding energy calculations, identified three promising candidates for SpFabG inhibition.

The review by Li et al. are discussed recent advances in the understanding of the relationships between structure, function, and allosteric regulation of the tryptophan synthase (TS) complex from *Salmonella typhimurium.* The synthesis of L-tryptophan (L-Trp) in enteric bacteria initiates at the level of gene expression by controlling the expression of the five enzymes of the Trp operon responsible for the L-Trp synthesis by the L-Trp cellular concentration. Two of these enzymes, trpA and trpB, form the αββα TS complex which is responsible for the last two enzymatic processes in the L-Trp synthesis. The authors details all the steps in which the TS system is involved and argue that the complex provides an unusual example of allosteric control.

Finally, Xu et al. determined the structures of enolase enzymes from *Mycoplasma pneumoniae* and *Mycoplasma bovis*. Mycoplasmas are a genus of bacteria that lack a cell wall, and its cell membrane peptidoglycan layer and are thought to be constantly in a rapid evolution. Enolases are enzyme central in glycolysis. The authors found that *mycoplasma* enolases contain an extra helix that contains F-K/G-K-L/F-K-X-A-I motifs. Comparison between the two enolase enzymes suggested a divergent evolution of *mycoplasma* enolases. Overall, these results provided the first direct proof of a rapid and divergent evolution of *mycoplasma* enolases. This study will help understanding the independent evolution of *Mycoplasma* species after separation from a common ancestor.

Overall, the six papers of this RT provide an overview of the most recent achievements made in the ever growing field of Structural Biology.
