# Correction: Editorial: Structural studies of bacteria and viruses

**DOI:** 10.3389/fmolb.2025.1697351

**Published:** 2025-09-08

**Authors:** Frontiers Production Office

**Affiliations:** Frontiers Media SA, Lausanne, Switzerland

**Keywords:** bacteria, virus, protein dynamic, pathogen biology, host interaction

The title of this article was erroneously given as: Editorial: Structural studies of bacterial and viruses. The correct title of the article is Editorial: Structural studies of bacteria and viruses.

The original article has been updated.

